# Perioperative Allogenenic Blood Transfusion Is Associated with Worse Clinical Outcomes for Hepatocellular Carcinoma: A Meta-Analysis

**DOI:** 10.1371/journal.pone.0064261

**Published:** 2013-05-31

**Authors:** Lei Liu, Zhiwei Wang, Songqi Jiang, Bingfeng Shao, Jibing Liu, Suqing Zhang, Yilong Zhou, Yuan Zhou, Yixin Zhang

**Affiliations:** 1 Department of Surgery, The Affiliated Tumor Hospital of Nantong University, Nantong, Jiangsu Province, China; 2 Department of Surgery, Renji Hospital, Shanghai Jiao Tong University School of Medicine, Shanghai, China; Cardiff University, United Kingdom

## Abstract

**Background and Objective:**

The impact of perioperative allogenenic blood transfusion (ABT) on clinical outcomes for hepatocellular carcinoma (HCC) is conflicting and unclear. The aim of this meta-analysis is to evaluate the association between ABT and HCC clinical outcomes. Outcomes evaluated were all-cause death, tumor recurrence and postoperative complications.

**Methods:**

Relevant articles were identified through MEDLINE search (up to November 2012). Meta-analyses were performed by using the fixed or random effect models. Study heterogeneity was assessed by Q-test and I^2^ test. Publication bias was evaluated by funnel plots, Egger′s and Begg’s test.

**Results:**

A total of 5635 cases from 22 studies finally met our inclusion criteria. Meta-analysis indicated HCC patients with ABT had an increased risk of all-cause death at 3 and 5 years after surgery (respectively: OR = 1.92, 95% CI, 1.61–2.29,P<0.001; OR = 1.60, 95% CI, 1.47–1.73,P<0.001 ) compared with those without ABT. The risk of tumor recurrence was significantly higher for ABT cases at 1, 3 and 5 years (respectively: OR = 1.70, 95% CI, 1.38–2.10, P<0.001; OR = 1.22, 95% CI, 1.08–1.38, P<0.001; OR = 1.16, 95% CI, 1.08–1.24, P<0.001). The HCC cases with ABT significantly increased postoperative complications occurrence compared with non-ABT cases (OR = 1.78,95% CI, 1.34–2.37, P<0.001).

**Conclusions:**

The findings from the current meta-analysis demonstrated that ABT was associated with adverse clinical outcomes for HCC patients undergoing surgery, including increased death, recurrence and complications. Therefore, ABT should not be performed if possible.

## Introduction

Hepatocellular carcinoma (HCC) is one of the most common malignancies all over the world, especially in Asia and Africa [1]. Although liver transplantation has already been proven as an alternative treatment method for HCC during the early stage, the scarcity of donors limits this treatment [2]. Hepatectomies is still the mainstay of treatment for patients with HCC. Most patients with HCC have poor hepatic function reserve. What is more, patients with liver cirrhosis showed decreased platelet count, which results in an increased risk of hemorrhage and prothrombin activity during surgery. Over the past decades, surgical techniques, perioperative care and increased experience have improved the safety of liver resection for HCC [3]. Despite these advances, partial liver resection still carries the risk for excessive blood loss and a subsequent need for blood transfusion. Blood transfusion rate during hepatic resections have been reported to be decreased from 62% in 1985 to 22% in 2007 [4].

Blood transfusion could save patients’ life once substantial hemorrhage occurred. The type of blood transfusion could be divided into autotransfusion and allogenenic blood transfusion (ABT). Autotransfusion represents autologous collection and reinfusion of patients’ own blood or blood components before surgery, while ABT refers to receive blood from other persons. However, ABT has been reported to be associated with potentially devasting complications, such as transmission of human immunodeficiency virus and hepatitis, transfusion reactions and increased postoperative infection rate [5]. Noninfectious risks such as transfusion-associated circulatory overload and transfusion-related acute lung injury (TRALI) are also well known[6]; [7]. There are several studies focused on the long-term outcomes after ABT in patients undergoing curative resection of HCC. Hanazaki et al [8] have reported that there were adverse effects of ABT on cancer recurrence and survival rate for HCC. Whereas, other investigators gave the inconsistent results. Kwon and his colleagues [9] demonstrated that ABT has no influence on the long-terms oncological outcome for HCC.

It has been seen that the association between ABT and clinical outcomes for HCC remains controversial. The published results were largely based on a retrospective analysis of cases from a single center, which could contribute to this inconsistent phenomenon. The method of meta-analysis could fully solve this problem, which played an important role in Evidence-medicine [10]. The rationale for a meta-analysis is that, by combining the samples of individual studies, the overall sample size is increased, thereby improving the statistical power of the analysis as well as the precision of the estimates of treatment effects [11]. As a result, on the basis of relevant literature search, we performed a comprehensive meta-analysis to fully estimate the postoperative influence of ABT for patients with HCC.

## Methods

This meta-analysis was done in accordance with the Preferred Reporting Items for Systematic Reviews and Meta-analyses (PRISMA) guideline [12].

### Identification and Eligibility of Relevant Studies

Studies were identified via a systematical search of the electronic database Pubmed (since 1967). The last search was performed on November 30^th^, 2012. The following keywords were used: “Hepatocelluar carcinoma (liver cancer)” and “blood transfusion”. And our search was restricted to articles published in English. Relevant lists of all relevant publications were hand searched for additional studies missed by the search strategy. To be included in our meta-analysis, the papers should meet all of the following criteria: (1) The studies had to evaluate the association between ABT and clinical outcomes (postoperative complications, recurrence or death) for HCC. (2) The report contained original data. (3) The papers focused on non-primary hepatocelluar carcinoma (e.g. benign tumors and metastatic neoplasm) or autogenic blood transfusion were excluded. (4) Reviews and letters to editors were not included because of insufficient data for analysis. If the same population was examined in the multiple studies, only the most recent report was included in the analysis.

### Outcomes

The primary outcome measures were postoperative complications, postoperative occurrences of tumor recurrence and all-cause death. The predefined time points for recurrence and all-cause death was three-year and five-year after surgery. Other time points such as one-year or ten-year could also be taken into account. The cause of death, if available, was analyzed for assessment of cancer-related mortality.

### Data Abstraction and Quality Assessment

In our study, we divided all cases into ABT group and non-ABT group. The ABT group was defined as cases who received any amount of allogenenic blood products (packed red blood cells, plasma, platelets or whole blood). And the non-ABT group was defined as cases who did not receive any blood products at all. The perioperative period included 30 days before and after surgery. Data abstraction was independently run by two of the authors (Liu and Wang). If they generated different results, a third investigator (Zhang) was asked to discuss to reach an agreement. Information retrieved from the original reports included first author’s name, publication year, study design, patients’ age and sex, liver cirrhosis, Child-Pugh class, tumor stage, amount of blood transfusion, number of patients included in the ABT and non-ABT group, complication and survival data for patients with and without ABT respectively.

The quality of each included study was assessed using the 9-star Newcastle-Ottawa Scale (Table S2). This scale is an eight-item instrument that allows for assessment of population and selection, study corporality, follow-up, and outcome of interest. Interpretation of the scale is performed by awarding points. Points are then added up, and each study could receive a score from 0 point (the lowest quality) to 9 points (the highest quality). High quality studies were defined as a study with a quality score more than 7 points, otherwise the studies were rated as poor quality.

### Statistical Analysis

For each outcome, odds ratio (OR) and its 95% confidence intervals (CI) was used to measure the association for each study. In this analysis, we examined the possible heterogeneity between studies using the X^2^-based Q test [13]. Where there was evidence of heterogeneity between studies (the P value for Q test less than 0.05), the random-effect model (the DerSimonian and Laird method) was used to calculate summary estimate; other wise, the fixed-effect model (the Mantei-Haenszle method) was applied [14]. Meanwhile, I^2^-test was also utilized to examine the heterogeneity (values of 25%, 50% and75% were considered to represent low, medium, and high heterogeneity respectively) [15]. The potential source of heterogeneity was evaluated by meta-regression (publication year, study design, sample size and sample constitution (the proportion of patients who underwent blood transfusion in each study)). Stratified analysis centered on potential confounders was also performed to evaluate the robustness of the conclusion drawn. Additionally, sensitivity analyses were carried out by excluding the highest or lowest OR value to assess the stability of the association between ABT and cancer clinical outcomes. Publication bias was assessed by funnel plots [16]. And the symmetry of funnel plots was analyzed using Egger’s and Begg’s test. All analyses were conducted using STATA 11.0 statistical software (Stata Corp, College Station, TX,USA).

## Results

### Literature Search and Study Characteristics

**Figure 1:**
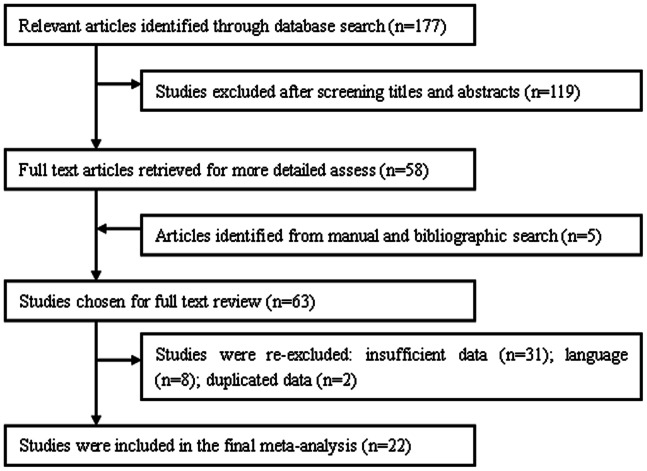
Flow diagram of the selection and screening process for eligible studies.

Figure 1 shows a flow diagram of how we selected relevant articles. The abstract of 177 primary studies were identified for initial review using the above-mentioned search strategies. After screening the abstracts, 58 full-text articles were retrieved for more detailed assess. Meanwhile, the hand search identified a further 5 articles. Eight were eliminated because of published language not in English, and thirty-one studies were also excluded due to duplication of data or inadequate data for analysis. Only one study reported the cancer-related survival data, it was impossible for us to adopt it to perform survival analysis. In total, 22 studies[8]; [9]; [17]; [18]; [19]; [20]; [21]; [22]; [23]; [24]; [25]; [26]; [27]; [28]; [29]; [30]; [31]; [32]; [33]; [34]; [35]; [36] met our inclusion criteria and were included in the final meta-analysis. Study characteristics were listed in Table 1.

**Table 1:** Characteristics of studies about ABT and hepatocellular carcinoma included in the meta-analysis.

Author	PublicationYear	Study type	Number of patients			ABTRate (%)	Age(mean,Y)	Sex(male%)	LiverCirrhosis(%)	Child A(%)	TNM stage		TransfusedUnits	Study quality
			ABT+	ABT−	Total						I+II	III+IV		
Matsumata et al^16^	1993	RCS	54	72	126	42.86	57.6	83.3	56.5					9
Itasaka et al^17^	1995	PCS	38	33	71	53.52	58.1	85.9	90.1	94.4			9.9	9
Yamamoto et al^18^	1996	RCS	85	301	386	22.02	59.7	80.6	59.3	68.7				6
Asahara et al^19^	1999	RCS	23	152	175	13.14	60.1	76.0	70.3		75	56		8
Makino et al^20^	2000	RCS	117	78	195	60.00	60.8	80.0	71.3	67.7				9
Kitagawa et al^21^	2001	PCS	26	52	78	33.33	60.0		55.5					7
Kwon et al^9^	2001	PCS	53	55	108	49.07	62.0	76.9	48.2	80.0	108	0	5.3	9
Ercolani et al^22^	2002	RCS	71	153	224	31.07	62.5	81.3	78.6	82.6				6
Sasaki et al^23^	2002	PCS	23	52	75	30.67	61.7	78.7		61,3	55	20		5
Wei et al^24^	2003	RCS	45	110	155	29.03	52.0	78.7	34.9				3.5	6
Hanazaki et al^8^	2005	RCS	210	158	368	57.07	62.4	75.6	52.2	78.3	300	68		9
Laurent et al^25^	2005	RCS	36	72	108	33.33	64.0	82.4	63.0		68	40		6
Sasaki et al^26^	2006	RCS	184	233	417	44.12		74.2	67.9	74.2	339	78		8
Sugita et al^27^ *	2008	RCS	101	120	221	45.70	63.2	77.0	54.5				7.9	9
Kaibori et al^28^	2008	RCS	269	141	410	65.61	64.2	79.8	39.5		264	136	5.0	9
Wang et al^29^	2009	RCS	62	411	473	13.11	53.1	80.1	44.4	83.3	400	73		8
Choi et al^30^	2009	RCS	91	96	187	48.66	51.0	75.4	56.7		99	88		6
Abdel-Wahab et al^31^	2010	RCS	87	72	159	54.72	54.8	75.0		87.0	138	37	3.0	7
Nanashima et al^32^	2011	RCS	100	83	183	54.64	65.0	80.9						7
Okamura et al^33^	2011	RCS	87	289	376	23.14	61.4	80.6		94.2	213	163		7
Yang et al^34^	2011	RCS	164	141	305	53.77	49.2	91.5	76.7	88.5	117	188	5.8	7
Kuroda et al^35^	2012	RCS	76	759	835	9.10	63.5	76.9	51.6	85.4			4.2	9

ABT+, allogenenic blood transfused; ABT−, non-allogenenic blood transfused; RCS, retrospective cohort study; PCS, prospective cohort study.

* Study provided cancer-related death data and could not be included.

All included studies gave a total of 5635 patients, of whom 2002 (35.5%) cases received ABT and 3633 (64.5%) cases were grouped as non-ABT. The sample size for each study varied from 71 to 835 (median: 175). The reported ABT rate in each article ranged from 9.10% to 65.61%. The average of all patients in all included studies was 61 years. The total proportion of male subjects was around 80% in all included studies. Of the 22 studies, 18 reported the presence or absence of liver cirrhosis, with approximately 60% of patients having liver cirrhosis. Twenty-two eligible studies comprised 4 prospective studies and 18 retrospective studies. Outcomes reported in each article included all-cause death (n = 12), tumor recurrence (n = 16) and postoperative complications (n = 7). All included studies were published between 1993 and 2012, of which 60% (n = 14) were published in 2007 or more recent years. According to the 9-star Newcastle-Ottawa Scale, half (n = 11) of the studies were defined as high-quality studies (score more than 7).

### All-cause Death

**Figure 2:**
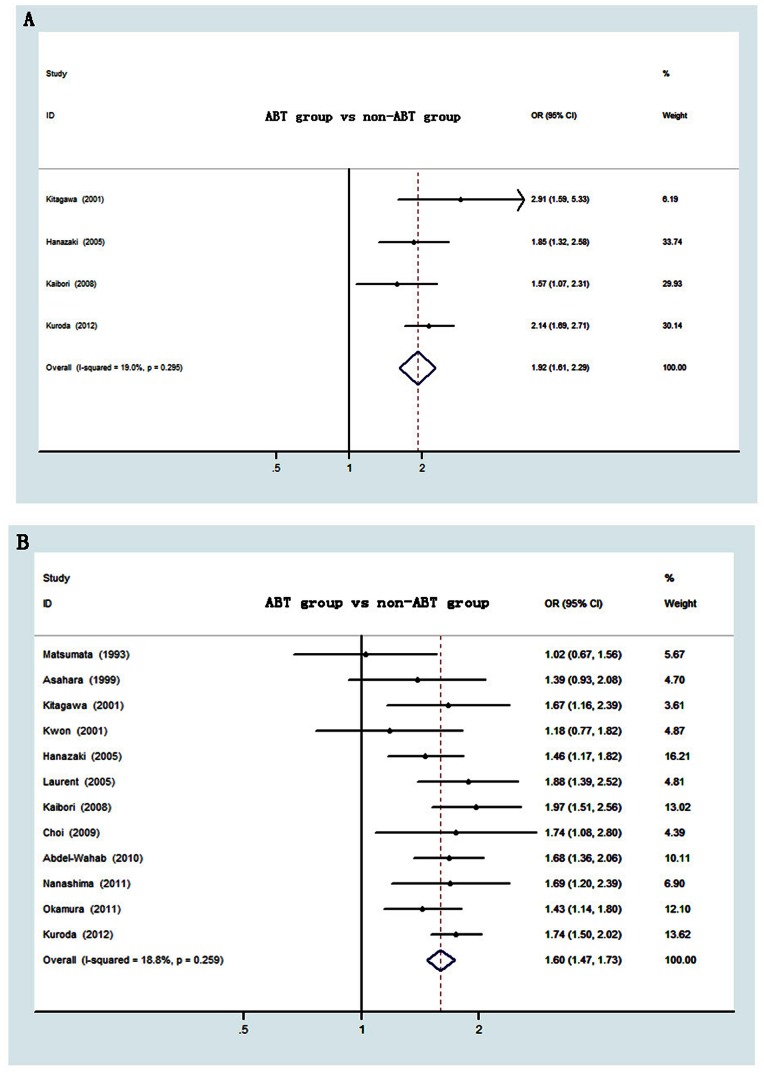
Forest plot of postoperative death risk associated with ABT for HCC. (**A**) for 3-year and (**B**) for 5-year. OR, odds ratio; CI, confidence interval.

Twelve studies reported the all-cause death, giving a total sample size of 3113 patients for evaluation. Of them, 4 studies[8]; [22]; [29]; [36] provided postoperative 3-year survival data and 12 studies[8]; [9]; [17]; [20]; [22]; [26]; [29]; [30]; [31]; [32]; [33]; [34]; [35]; [36] provided 5-year survival data. As shown in Figure 2A, meta-analysis data showed a significant higher death risk for patients with ABT than those without ABT during the 3 years after surgery, corresponding to a pooled of OR of 1.92 (95% CI:1.61–2.29, P<0.001). Between-study heterogeneity was not observed (I^2^ = 19.0%, P = 0.295). Regarding to postoperative 5-year death risk, meta-analysis of all studies demonstrated a significant increased all-cause death after ABT, with an overall OR of 1.60 (95% CI:1.47–1.73, P<0.001) (Figure 2B). Significant heterogeneity among studies was not present (I^2^ = 18.8%, P = 0.259).

Table 2 shows the results of the stratified meta-analysis for postoperative 5-year all-cause death. When stratified by sample size, ABT could increase the postoperative all-cause death risk in large sample-size studies with the overall estimate (OR = 1.65, 95% CI,1.49–1.83, P<0.001). Similar results were obtained in small sample-size studies (OR = 1.48, 95% CI,1.30–1.69, P<0.001). By sample constitution, ABT was associated with increased risk of death in low ABT rate group (OR = 1.59, 95% CI,1.38–1.82, P<0.001) and high ABT rate group (OR = 1.60, 95% CI,1.44–1.77, P<0.001). Studies with high quality (OR = 1.71, 95% CI,1.51–1.93, P<0.001) demonstrated slightly increased death risk than those with poor evidence level (OR = 1.50, 95% CI,1.29–1.73, P<0.001). When examining differences over time, we found that studies published before 2007 had a summary estimate with a pooled OR of 1.71 (95% CI,1.54–1.90, P<0.001 ), while studies published after 2007 had an overall estimate with a pooled OR of 1.45 (95% CI, 1.23–1.72, P<0.001 ). Sensitivity analysis was done for postoperative 5-year all-cause death by excluding the studies with the highest and the lowest OR value[17]; [29] and analyzing the impact on the results. The result did not alter the overall effect, giving a pooled OR of 1.58 (95% CI 1.45–1.72, P<0.001). Meanwhile, heterogeneity tests between subgroups were also performed. The results showed no heterogeneity was observed (P>0.05) (Table 2).

**Table 2:** Stratified analyses for 5-year all-cause death.

Groups	No. studies	Meta-OR		Heterogeneity		Heterogeneity between subgroup
		95% CI	P-value	I^2^ (%)	P-value	P-value
All studies	12	1.60 (1.47–1.73)	<0.001	18.8	0.259	
Sample size#						0.434
Studies with ≤179 cases	6	1.48 (1.30–1.69)	<0.001	40.0	0.139	
Studies with >179 cases	6	1.65 (1.49–1.83)	<0.001	0	0.419	
Sample constitution#						0.371
Low transfusion rate	4	1.59 (1.38–1.82)	<0.001	0	0.744	
High transfusion rate	8	1.60 (1.44–1.77)	<0.001	42.9	0.092	
Study quality						0.762
Low quality	7	1.50 (1.29–1.73)	<0.001	37.2	0.145	
High quality	5	1.71 (1.51–1.93)	<0.001	0	0.484	
Publication year						0.084
Before 2007	6	1.71 (1.54–1.90)	<0.001	0	0.622	
After 2007	6	1.45 (1.23–1.72)	<0.001	30.6	0.206	

# Median number of patients included: 179.

## Mean transfusion rate of included studies: 40.4%.

### Tumor Recurrence

**Figure 3:**
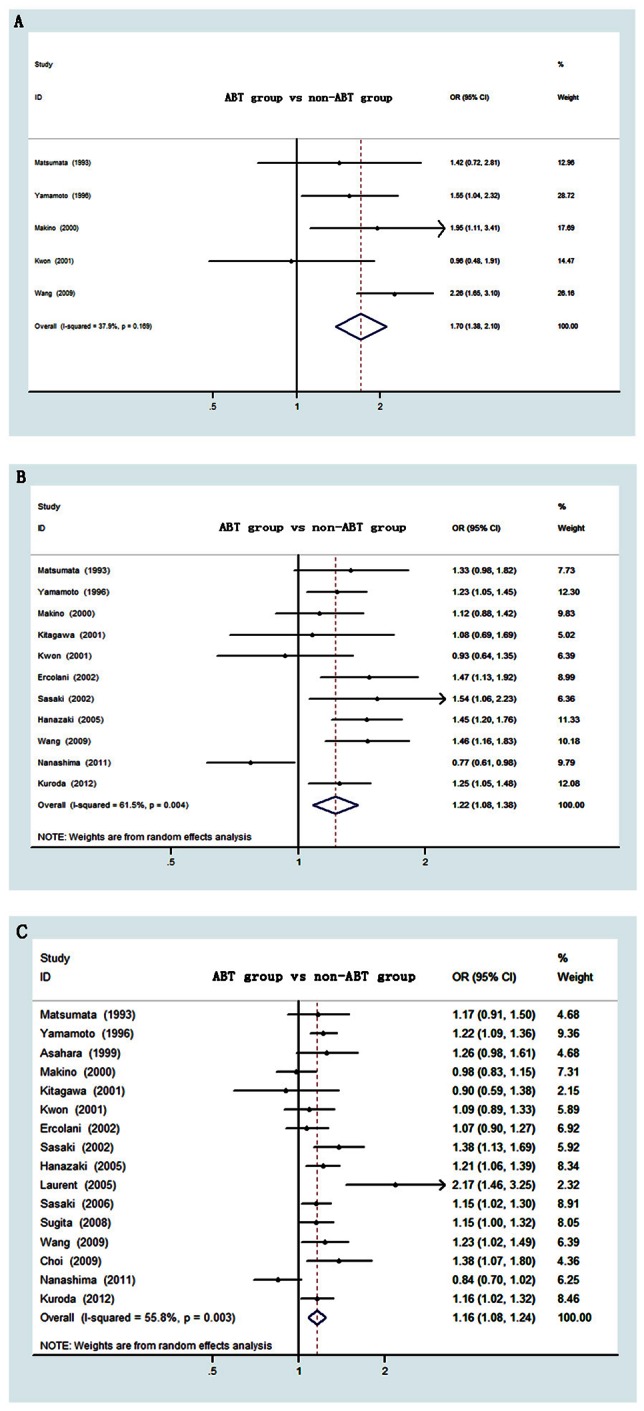
Forest plot of postoperative recurrence risk associated with ABT for HCC. **(A)** for 1-year, **(B)** for 3-year and **(C)** for 5-year. OR, odds ratio; CI, confidence interval.

Sixteen studies reported the data of disease progression defined as recurrence in ABT and non-ABT patients, involving 4120 cases. Postoperative 1-year data were available for five studies[9]; [17]; [19]; [21]; [30], 3-year data were reported by eleven studies[8]; [9]; [17]; [19]; [21]; [22]; [23]; [24]; [30]; [33]; [36] and 5-year data were provided by sixteen studies[8]; [9]; [17]; [19]; [20]; [21]; [22]; [23]; [24]; [26]; [27]; [28]; [29]; [30]; [31]; [32]; [33]; [34]; [35]; [36]. As displayed in Figure 3A, the result of meta-analysis suggested a significantly increased risk of recurrence for patients with ABT during the one year after surgery (OR = 1.70, 95% CI 1.38–2.10, P<0.001). Significant heterogeneity did not exist among these studies (I^2^ = 37.9%, P = 0.169), hence the fixed effect module was adopted.

With regard to the 3-year risk after operation, the overall estimate also showed patients with ABT had a significantly increased risk of recurrence than those without ABT (OR = 1.22, 95% CI 1.08–1.38, P<0.001) (Figure 3B). However, significant heterogeneity between studies was observed (I^2^ = 61.5%, P = 0.004), and the random effect module was applied.

As for the postoperative 5-year risk of recurrence, the meta-analysis indicated patients with ABT had a significantly increased risk compared with non-ABT patients, giving a summary OR of 1.16 (95% CI 1.08–1.24, P<0.001) (Figure 3C). The random effect module was implemented due to slightly significant heterogeneity across the studies (I^2^ = 55.8%, P = 0.003). A meta-regression was performed to evaluate the potential source of heterogeneity, which regressed the parameters of publication year, sample size, study design and sample constitution. Unfortunately, we failed to detect any factor that was recognized as the main source of between-study heterogeneity (P>0.05) (data not shown). Sensitivity analysis was carried out by exclusion of the studies with the highest and the lowest OR value [8]; [33]. Nearly no changes of the pooled OR was observed (OR = 1.17, 95% CI 1.12–1.22, P<0.001).

### Postoperative Complications

**Figure 4:**
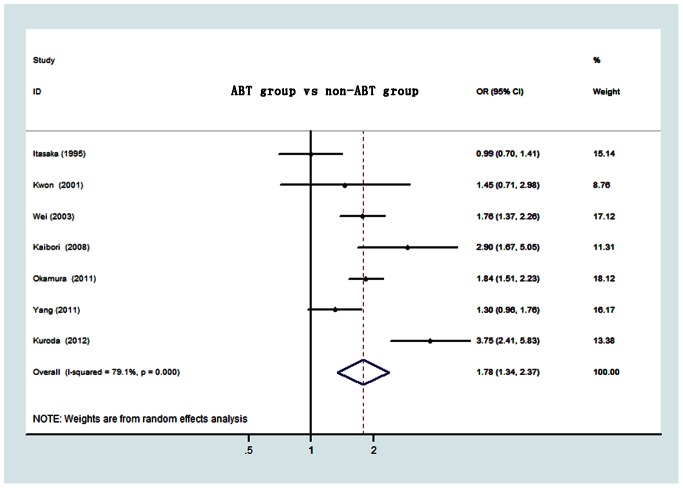
Forest plot of postoperative complications risk associated with ABT for HCC. OR, odds ratio; CI, confidence interval.

Seven studies examined the relationship between ABT and postoperative complications, which enrolled 2260 patients. The combined OR of postoperative complications based on these studies was 1.78 (95% CI:1.34–2.37, P<0.001)(Figure 4). Significant heterogeneity among studies was present (I^2^ = 79.1%, P<0.001). Because of limited number of studies, sensitivity analysis was not able to be carried out in our study.

### Publication Bias

**Figure 5:**
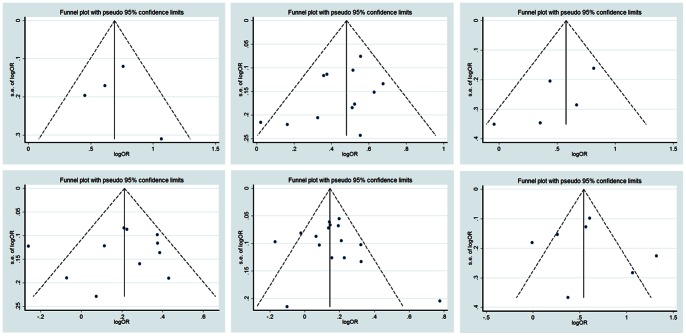
Funnel plot of publication bias test for ABT and HCC clinical outcomes. Postoperative death at 3 year **(upper left)**, postoperative death at 5 year **(upper middle)**, postoperative recurrence at 1 year **(upper right)**, postoperative recurrence at 3 year **(lower left)**, postoperative recurrence at 5 year **(lower middle)**, postoperative complications **(lower right)**. Horizontal axis represents the log of OR; vertical axis represents SE of log(OR). CI, confidence interval; OR, odds ratio; Log, logarithm; SE, standard error.

We used Egger’s test and Begg’s test to evaluate the publication bias. No indication of publication bias was observed for all-cause death at 3 year (Egger’s test, P = 0.876; Begg’s test, P = 1.000), and at 5 year (Egger’s test, P = 0.183; Begg’s test, P = 0.115); for tumor recurrence at 1 year (Egger’s test, P = 0.130; Begg’s test, P = 0.142), at 3 year (Egger’s test, P = 0.682; Begg’s test, P = 0.533), and at 5 year (Egger’s test, P = 0.590; Begg’s test, P = 0.882); for postoperative complications (Egger’s test, P = 0.747; Begg’s test, P = 0.881). The funnel plots for the relation between ABT and the clinical outcomes of HCC are shown in Figure 5.

## Discussion

The possibility to store and transfuse blood has been a major advance in medicine in the 20^th^ century, saving countless lives. Nevertheless, the relationship between ABT and tumor clinical outcomes has been debated in the literature for several decades. A number of authors have reported that ABT was associated with deleterious outcomes for HCC. For instance, in a series of 183 patients undergoing hepatectomy for HCC, Hanazaki et al [8] have demonstrated that ABT was a risk factor for tumor recurrence and worse prognosis. Whereas, Kwon and his colleagues [9] reported no effect of ABT on survival after hepatic resection. There is a lack of systematic evaluation focused on ABT for HCC. Therefore, we comprehensively searched through PubMed for all medical literature published in English-language journals until 2012 and performed a meta-analysis of the relationship between ABT and HCC clinical outcomes.

To our knowledge, this study provided the first comprehensive meta-analysis of effect of ABT on clinical outcomes after surgery for HCC. Twenty-two studies with a combined patient population of more than 5000 patients during the past two decades all over the world met the criteria and were included in the final meta-analysis. Our meta-analysis illustrated that ABT in patients undergoing surgery for HCC was associated with significantly worse outcomes, including all-cause death, tumor recurrence and postoperative complications. The meta-OR for all-cause death during the five years after surgery was 1.60 in this study. It means that patients with ABT had a 60% increased death risk than those without ABT. In terms of postoperative tumor recurrence, the combined OR value for five years was 1.16 in our study. That is to say, compared with patients without ABT, those with ABT had a more than 16% chance of tumor recurrence. Similar adverse effect of ABT on clinical outcomes was also observed in other malignancies. For instance, a recently published meta-analysis based on 20795 cases has proven that ABT was significantly associated with increased all-cause death (OR = 1.72, 95% CI:1.55–1.91, P<0.001) and more tumor recurrence (OR = 1.66, 95% CI:1.41–1.97, P<0.001) for colorectal cancer patients [37]. Meanwhile, this study suggested ABT was also associated with other adverse outcomes, such as postoperative infections, need for surgical reintervention and increased length of hospital stay. Another previous meta-analysis performed by Yao and his colleagues on ampullary cancer suggested that interoperative ABT was also associated with worse prognosis (OR = 2.55, 95% CI: 1.59–4.10) [38].

Our study demonstrated a consistent survival disadvantage of ABT in different types of analyses. What’s more, subgroup analysis by sample size, sample constitution, evidence level and publication year all displayed the similar survival disadvantage. And sensitivity analysis by excluding the outlying studies was performed and we noted a similar worse prognosis. Moreover, all of our meta-analysis for all-cause death and tumor recurrence at different time after surgery gave the consistent results. The uniform of these adverse outcomes across the spectrum of analyses demonstrated the validity and robustness of the meta–analysis. Interestingly, the postoperative risk of death and recurrence was gradually decreased over time. It may be seen that the impact of ABT on HCC clinical outcomes was greater in short term after surgery.

The reason why ABT can cause worse clinical outcomes remains uncertain. And the mechanism underlying the adverse effects of blood transfusion has been assumed to be related to the suppressive effects on the immune system. Several investigators have reported that blood transfusion suppress host immunity via a reduction in T lymphocytes function, decreased natural killer cell function, increased numbers of T-suppressor cells and decreased function of macrophages and monocytes[39]; [40]; [41]. In recent years, an increasing body of studies have demonstrated that several lymphocyte surface markers were significantly changed after ABT, and they were closely related with tumor proliferation, apoptosis and progression to metastasis. These studies have shown that the CD2 and CD4 level were decreased during postoperative period, as compared with non-transfused patients[42]; [43]. In addition, other studies have suggested that soluble HLA class I and soluble Fas-ligand released by leukocytes present in blood products inhibit the activity of NK cells and cytotoxic T cells, which are known to reduce immune capacity, and may predispose to postoperative infections[44]; [45]; [46].

Our study has other advantages except for the consistency of overall results. Up to now, the number of cases included in our study is one of the largest. Although publication bias is one of the major drawbacks of any meta-analysis, no publication bias was detected by Egger’s and Begg’s test, which indicated that statistical results approximated the truth. However, several limitations should be noted in our study. Firstly, all the included articles were non-randomized studies, and the evidence level is lower than that of randomized controlled trial (RCT). One reason for this is that there is no choice for patients with substantial hemorrhage but transfusion. Hence, this needs to be kept in mind when interpreting our result. In the absence of RCT now or in the future, this meta-analysis may represent a comprehensive analysis of the impact of ABT for patients with HCC. Secondly, the characteristics of patients may differ between the studies. As we know, the factors impacted the clinical outcomes of HCC patients after surgery include hepatitis C infection, pre-treatment α-fetoprotein level, liver cirrhosis and tumor stage[47]; [48]. Because of limited information, we were not able to control these potential confounded factors. Thirdly, surgery-related risk factors such as type and duration of operation should not be neglected. Postoperative transcatheter arterial chemoembolization (TACE) has been involved significantly over the past years, with significant improvement in survival[49]; [50]. The adjuvant treatment received by each patient was not fully understood, which could lead unknown effect to our results. Meanwhile, the lack of individual case data limited our ability to analyze the cancer-related death risk in our study. Future studies should prospectively record these data. Last but not least, although there was no evidence of significant heterogeneity for all-cause death analysis, slightly significant heterogeneity was found in the meta-analysis of recurrence and complications in our study. Regretfully, meta-regression could not explain any source, which reminded us some underlying factors may contribute to the heterogeneity. The exclusion of studies published in languages other than English was another potential limitation to our meta-analysis. It is well known that Eastern Asia and Sub-Saharan Africa were high risk areas for HCC and some articles were not published in English. Hence, these studies focused on this specific issue would not meet our inclusion criteria for our meta-analysis. These studies can have little bearing on the results of this meta-analysis, although there was no publication bias in our study.

In conclusion, this meta-analysis has demonstrated that ABT has a significantly deleterious effect on clinical outcomes of HCC patients, and is associated with an elevated risk of death, recurrence and postoperative complications. Theses findings emphasize the need for meticulous surgical technique to minimize blood loss. Therefore, to achieve a better outcome of HCC patients undergoing liver resection, ABT should not be performed if possible. In addition, the use of artificial blood products or marrow stimulants such as erythropoietin should be advocated fully. Meanwhile, the results of our study need to be interpreted in the backdrop of the subjective design of the meta-analysis with its inherent advantages and disadvantages. As a result, further randomized controlled trails would be better suited to address this issue exactly.

## Supporting Information

Table S1 **Checklist of items to include when reporting a systematic review or meta-analysis.**
(DOC)

Table S2 **Newcastle-Ottawa quality assessment scale.**
(DOC)
