# Supplementary material for: Perioperative Allogenenic Blood Transfusion Is Associated with Worse Clinical Outcomes for Hepatocellular Carcinoma: A Meta-Analysis
Source: PLoS One. 2013 May 31;8(5):e64261. doi: 10.1371/journal.pone.0064261 (PMC3669337; doi:10.1371/journal.pone.0064261)
Supplement: Table S2 — Newcastle-Ottawa quality assessment scale. (DOC) [file pone.0064261.s002.doc]

**Table S2 Newcastle-Ottawa quality assessment scale^※^**

**Selection**

(1) Representativeness of the exposed cohort

(a) Truly representative of the average‘HCC patient’ in the community (1 star)

(b) Somewhat representative of the average‘HCC patient in the community(1 star)

(c) Selected group of users (e.g. nurses, volunteers)

(d) No description of the derivation of the cohort

(2) Selection of the non-exposed cohort

(a) Drawn from the same community as the exposed cohort (1 star)

(b) Drawn from a different source

(c) No description of the derivation of the non-exposed cohort

(3) Ascertainment of exposure (Proof of HCC and blood transfusion)

(a) Secure record (eg surgical records) (1 star)

(b) Structured interview (1 star)

(c) Written self-report

(d) No description

(4) Demonstration that outcome of interest was not present at start of study

(a) Yes (1 star)

(b) No

**Comparability**

(1) Comparability of cohorts on the basis of the design or analysis

(a) Study controls for confounder factor (Age, sex)(1 star)

(b) Study controls for any additional factor (1 star) (Child class, stage, tumor size etc.)

**Outcome**

(1) Assessment of outcome (Death or recurrence)

(a) Independent blind assessment (1 star)

(b) Record linkage (1 star)

(c) Self-report

(d) No description

(2) Was follow-up long enough for outcomes to occur? (Death or recurrence)

(a) Yes (‘5years’) (1 star)

(b) No

(3) Adequacy of follow-up of cohorts

(a) Complete follow-up – all subjects accounted for (1 star)

(b) Subjects lost to follow-up unlikely to introduce bias – small number lost‘(25%)’ or description provided of those lost (1 star)

(c) Follow-up rate less than ‘75%’ and no description of those lost

(d) No statement

**^※^**Wells GA, Shea B, O'Connell D, Peterson J, Welch V et al. The Newcastle-Ottawa Scale (NOS) for assessing the quality of nonrandomized studies in meta-analyses. Ottawa Hospital Research Institute. <http://www.ohri.ca/programs/clinical_epidemiology/oxford.asp>.
